# CARDIOVASCULAR RISK FACTORS IN DIABETIC INTERNET USERS – COMPARATIVE ANALYSIS

**DOI:** 10.13075/ijomeh.1896.02586

**Published:** 2025

**Authors:** Bartosz G. Trzeciak, Piotr Gutknecht, Szymon Grymek, Janusz Siebert

**Affiliations:** 1 Medical University of Gdańsk, Department of Family Medicine, Gdańsk, Poland; 2 Gdańsk University of Technology, Institute of Mechanics and Machine Design, Faculty of Mechanical Engineering and Ship Technology, Gdańsk, Poland

**Keywords:** hypertension, diabetes mellitus, coronary artery disease, hypercholesterolemia, cardiovascular risk factors, tobacco users

## Abstract

**Objectives:**

This study presents a comprehensive analysis of cardiovascular risk factors in individuals with diabetes mellitus compared to a non-diabetic control group, utilizing data from a risk program – the Ryzyko Program of the Medical University of Gdańsk, Gdańsk, Poland.

**Material and Methods:**

The research encompasses a sample of 2959 diabetic and 33 865 non-diabetic internet users. Variables such as gender, systolic blood pressure (SBP), total cholesterol (TC), smoking status, and the prevalence of coronary artery disease (CAD) were analyzed. Significant gender differences in smoking habits and cholesterol levels were observed in the diabetic group compared to the control group.

**Results:**

Diabetic individuals exhibited a higher prevalence of hypertension (SBP ≥140 mm Hg), with 58.7% of diabetic participants having poorly controlled hypertension. The study also reveals a higher incidence of CAD in the diabetic group, with a prevalence of 31.0%, compared to 8.3% in the control group. Notably, the diabetic group showed higher rates of cigarette smoking and elevated levels of arterial pressure, both in the entire group and across gender subgroups. The diabetic group demonstrated significantly increased SBP and TC levels compared to the non-diabetic control group as well as higher rates of CAD, and smoking in individuals with diabetes mellitus.

**Conclusions:**

These findings underscore the necessity for targeted cardiovascular risk management in the diabetic population.

## Highlights

- Internet-based health data shows the power of digital epidemiology.
- Gender-specific differences reveal tailored risk management needs.
- High prevalence of hypertension and coronary artery disease highlights critical areas for interventions.
- Findings stress targeted cardiovascular care for diabetics.
- Analysis of 36 824 participants ensures robust findings.

## INTRODUCTION

Cardiovascular diseases (CVDs) remain a leading cause of morbidity and mortality globally, being responsible for approx. 17.9 million deaths annually [1]. The burden of CVDs is exacerbated by the presence of various risk factors, such as older age, male sex, cigarette smoking, hypertension, hypercholesterolemia, and type 2 diabetes mellitus (T2DM) [2]. The latter has been increasingly recognized as a significant contributor to cardiovascular risk, affecting an estimated 536.6 million adults worldwide [3] and 2.9 million in Poland [4]. There is a strong association between diabetes and the risk of vascular diseases. A meta-analysis of 102 prospective studies demonstrated that diabetes and fasting glucose levels are significant risk factors for vascular diseases [5]. Understanding the interplay between T2DM and other cardiovascular risk factors is crucial for effective prevention and management strategies [6].

Recent studies have provided valuable insights into the role of T2DM in cardiovascular risk. For instance, a study by Sasso et al. [7] found that the number of uncontrolled risk factors was directly associated with cardiovascular outcomes in T2DM patients with albuminuria. Another study by Di Folco et al. [8] explored the effects of semaglutide on cardiovascular risk factors and eating behaviors in T2DM patients, revealing significant improvements in metabolic control. Furthermore, a comprehensive scientific statement from the American Heart Association emphasized the need for a multi-faceted approach to managing cardiovascular risk in adults with T2DM [9].

Analyses that compare cardiovascular risk profiles between T2DM and non-T2DM populations are essential for understanding the specific contributions of T2DM to cardiovascular risk and for tailoring prevention strategies accordingly [10].

The primary objective of this study is to conduct a comparative analysis of cardiovascular risk factors, including age, gender, smoking status, hypertension, and hypercholesterolemia, between individuals with diabetes and non-diabetic control group. The authors hypothesize that diabetes significantly modulates these risk factors, thereby contributing to an elevated risk of developing CVDs.

## MATERIAL AND METHODS

The website of the Medical University of Gdańsk, Gdańsk, Poland, offers access to the Ryzyko Program [11,12]. High-risk countries (such as Poland) utilize the Systematic Coronary Risk Evaluation (SCORE) algorithm to evaluate the risk for cardiovascular death over a 10-year period. This algorithm was published in the European Heart Journal [13].

In the current study, internet users with diabetes were analyzed, totaling 2959 individuals from 2004 until the time of publication. The control group (N = 33 865 individuals) consisted of internet users without diabetes from the Ryzyko Program database.

The study collected data on participants' age, sex, systolic blood pressure (SBP), total cholesterol (TC) concentration, and smoking status. Additional information regarding the diagnosis of diabetes or coronary artery disease (CAD) was also gathered. Individuals with diabetes are part of the high cardiovascular risk group regardless of their calculated SCORE algorithm result, as diabetes itself is a sufficient factor indicating their high-risk status.

Upon entering personal information as requested by the program, the participant could view both graphic and numerical outcomes. In cases where the results exceed the limits suggested by the European Society of Cardiology and Prevention, and/or the calculated risk of death is ≥5% over a 10-year period, it is advisable to visit a physician [13].

Individuals with diabetes are part of the high cardiovascular risk group regardless of their calculated SCORE algorithm result. Diabetes itself is a sufficient factor indicating their high-risk status. However, in the study, calculated risk results from the SCORE algorithm were also cited to illustrate and differentiate between groups.

All epidemiological data and results were gathered on a MySQL database hosted on the server of the Medical University of Gdańsk.

The results of the study are presented as a percentage of the relevant population, with numbers rounded to 1 or 2 decimal places (for TC mean values). Descriptive statistics were employed, considering a p-value <0.05 as statistically significant. For continuous variables, the Anderson-Darling test and the Lilliefors test were applied to confirm normal distribution in the samples. The Bartlett test and the Brown-Forsythe test were then used to assess the equality of variances in the compared groups. When the conditions for ANOVA analysis were not met, the Kruskal-Wallis test, a non-parametric alternative to ANOVA, was performed. This test also yields a p-value. For categorical variables, Pearson's χ^2^ test of independence, based on the contingency table, was utilized. The basic condition for its reliability was consistently met. This test is used to determine the likelihood of a relationship between variables and calculates a p-value.

This study employs a cross-sectional design, utilizing data from electronic health records to compare cardiovascular risk factors between diabetic and non-diabetic populations.

The article was prepared within the Medical University of Gdańsk framework of the institutional statutory project ST72. The study protocol was approved by the Bioethics Committee in Gdańsk on July 1, 2015 approval No. NKEBN/923/2004. No external funding was received.

Informed consent was not required for this study, as it was conducted retrospectively using data collected anonymously, with no possibility of identifying the individuals included in the analysis. The study design complied with the principles of the Declaration of Helsinki and relevant data protection regulations.

## RESULTS

In the observed diabetes group, there were 2072 men and 887 women. The average age was higher in the female group. Men reported smoking cigarettes more frequently compared to women. Elevated levels of TC (>5 mmol/l) were more common in the female group. Hypertension occurred at a similar level among both women and men – 59.0% and 58.5%, respectively. The prevalence of CAD was similar in both groups (Table 1).

**Table 1.** Internauts with diabetes – cardiovascular risk factors, data from a risk program – the Ryzyko Program of the Medical University of Gdańsk, Gdańsk, Poland, 2004–2025

Variable	Participants (N = 2959)			p
	total	male (N = 2072)	female (N = 887)	
Sociodemographic				
age [years] (M±SD)	55.0±11.7	54.2±11.2	56.9±12.6	<0.001^a^
Medical				
systolic blood pressure [mm Hg] (M±SD)	141.5±15.5	141.7±15.5	140.9±15.7	0.255^a^
hypertension [n (%)]	1736 (58.7)	1213 (58.5)	523 (59.0)	0.832^b^
total cholesterol [mmol/l] (M±SD)	5.82±0.97	5.8±0.99	5.85±0.93	0.211
hypercholesterolemia [n (%)]	2311 (78.1)	1596 (77.0)	715 (80.6)	0.031^b^
coronary artery disease [n (%)]	914 (31.0)	655 (31.6)	259 (29.2)	0.193^b^
Smoking [n (%)]	1177 (39.8)	868 (41.9)	309 (34.8)	<0.001^b^

Hypertension – systolic blood pressure ≥140 mm Hg.

Hypercholesterolemia – total cholesterol >5 mmol/l.

a The Kruskal-Wallis test.

b The χ^2^ test.

When compared to the control group, individuals with diabetes exhibited higher blood pressure across the entire group as well as within the subgroups of men and women. Additionally, the diabetes group showed higher rates of elevated levels of arterial pressure (SBP ≥140 mm Hg), cigarette smoking, and a greater prevalence of CAD. While cholesterol levels showed insignificant differences overall, a statistically significant difference was noted within the entire group and among men (Table 2).

**Table 2.** Risk factors comparison among diabetic vs. non-diabetic internauts (control group), data from a risk program – the Ryzyko Program of the Medical University of Gdańsk, Gdańsk, Poland, 2004–2025

Variable	Participants (N = 36 824)								
	total		p	male (N = 22 563)		p	female (N = 14 261)		p
	with diabetes mellitus	non-diabetic (control)		with diabetes mellitus	non-diabetic (control)		with diabetes mellitus	non-diabetic (control)	
Sociodemographic									
gender [n]	2959	33 865	<0.001	2072	20 491	n.a.	887	13 374	n.a.
age [years] (M±SD)	55.0±11.7	47.9±12.1	<0.001^a^	54.2±11.2	46.8±11.9	<0.001^a^	56.9±12.6	49.5±12.3	<0.001^a^
Medical									
systolic blood pressure [mm Hg] (M±SD)	141.5±15.5	135.8±14.0	<0.001^a^	141.7±15.5	136.6±13.9	<0.001^a^	140.9±15.7	134.5±14.0	<0.001^a^
hypertension [n (%)]	1736 (58.7)	14431 (42.6)	<0.001^b^	1213 (58.5)	9239 (45.1)	<0.001^b^	523 (59.0)	5192 (38.8)	<0.001^b^
total cholesterol [mmol/l] (M±SD)	5.82±0.97	5.77±0.93	0.006^a^	5.80±0.99	5.73±0.93	0.001^a^	5.85±0.93	5.83±0.93	0.443^a^
hypercholesterolemia [n (%)]	2311 (78.1)	26 284 (77.6)	0.542	1596 (77.0)	15 588 (76.1)	0.331	715 (80.6)	10 696 (80.0)	0.648
coronary artery disease [n (%)]	914 (31.0)	2804 (8.3)	<0.001^b^	655 (31.6)	1843 (9.0)	<0.001^b^	259 (29.2)	961 (7.2)	<0.001^b^
Smoking [n (%)]	1177 (39.8)	9351 (27.6)	<0.001^b^	868 (41.9)	6059 (29.6)	<0.001^b^	309 (34.8)	3292 (24.6)	<0.001^b^

n.a. – not applicable.

Hypertension – systolic blood pressure ≥140 mm Hg.

Hypercholesterolemia – total cholesterol >5 mmol/l.

a The Kruskal-Wallis test.

b The χ^2^ test.

## DISCUSSION

Diabetes is characterized by chronic hyperglycemia, which causes irreversible damage to blood vessels and consequently leads to macrovascular complications (CAD, stroke, peripheral arterial disease, and erectile dysfunction) and microvascular complications (retinopathy, nephropathy, and diabetic neuropathy) [14].

Metabolic control is crucial in preventing chronic complications of diabetes. Intensive diabetes treatment significantly reduces the risk of developing and progressing long-term complications in non-insulin-dependent diabetes [15].

Although there has been a recent decrease in cardiovascular complications, primarily due to the introduction of primary and secondary prevention programs and advances in the treatment of metabolic diseases, micro- and macroangiopathic complications still lower the quality of life and generate substantial treatment costs [9,16]. The increasing prevalence of diabetes presents a challenge for healthcare systems worldwide [17]. The occurrence of CVDs in middle-aged individuals reaches up to 41% in those with diabetes [18]. In this study, the prevalence of CAD was lower – 31.0%, similar in men and women and 4 times more frequent than in controls (Table 2).

One of the strongest cardiovascular risk factor is cigarette smoking. Internet users from the Ryzyko Program reported cigarette smoking in 41.9% of men and 34.8% of women with diabetes (Table 1). However, among women, the percentage of smokers was also higher than in the general European population. The prevalence of tobacco use in the European population was similar to that of the control group (Table 2). Approximately 1.3 billion people worldwide use tobacco products, with >80% residing in low- and middle-income countries. Tobacco contributes to the premature death of half of its users. The magnitude of the problem remains significant; in 2020, 22.3% of the global population used tobacco products (36.7% men and 7.8% women). In Europe, the estimated prevalence of tobacco use is 25.3% of the population.

It is important to note that not only direct tobacco users but also individuals exposed to secondhand smoke, which fills restaurants, offices, homes, or other enclosed spaces when people smoke tobacco products, have an increased risk of mortality [19]. This fact may influence the reported differences in the outcomes observed across various populations.

Hyperglycemia exacerbates the harmful effects of cigarette smoking, significantly increasing the risk of both microvascular and macrovascular complications in patients with T2DM [20]. Concurrently, there is evidence that cessation of cigarette smoking reduces the risk in these individuals [21]. Efforts to reduce cigarette smoking in the population with diabetes, alongside diabetes treatment, appear to be key according to the latest diabetes treatment guidelines [22].

The updated guidelines introduce a new category for individuals with diabetes who are considered to be at “extremely high cardiovascular risk” due to a recent acute coronary syndrome and the presence of ≥1 additional risk factor, such as elevated lipoprotein(a) (Lp(a)) levels >50 mg/dl, high-sensitivity C-reactive protein (hsCRP) >3 mg/l, or chronic kidney disease (defined as estimated glomerular filtration rate [eGFR] <60 ml/min/1.73 m^2^). For this patient subgroup, more stringent lipid targets are recommended: low-density lipoprotein cholesterol (LDL-C) <40 mg/dl (<1.0 mmol/l), non-high-density lipoprotein cholesterol (HDL-C) <70 mg/dl (<1.8 mmol/l), and apolipoprotein B (ApoB) <55 mg/dl (0.55 g/l) [22].

In this study, the authors did not determine the serum concentrations of cholesterol fractions. However, a study published in 2023 indicated that the level of TC can also serve as a therapeutic target in patients with diabetes to reduce cardiovascular risk [23].

Among internet users of the Ryzyko Program, elevated TC levels (>5 mmol/l) were more frequently observed in women, with a prevalence of 80.6%. A comparable proportion was noted in the overall control group – 80.0% (Table 2). These values exceed those reported in the WOBASZ study, where the prevalence of elevated TC was 64.3% in women and 70.3% in men [24].

The reasons for insufficient lipid level control may be linked to patients' concerns about complications from statin therapy and post-statin myalgia, but also possibly due to medical recommendations [25,26]. However, there are studies indicating that in patients with atherosclerosis, lowering LDL-C levels to a very low level of <20 mg/dl (<0.5 mmol/l) led to a reduction in cardiovascular incidents without complications related to the initiation of lipid-lowering therapy [27]. Other studies point to extracardiac benefits of statin use, such as improved bone metabolism [28].

Hypertension or elevated blood pressure is a key modifiable risk factor for CVD, and its control is paramount for CVD prevention [29]. Hypertension is associated with diabetes at a prevalence exceeding 70%, and it increases the probability of target organ damage and the incidence of clinical complications [30]. High blood pressure is associated with up to 13.5% of all deaths annually worldwide and is considered the leading risk factor for CVDs. Well-controlled SBP may offer the greatest potential among other risk factors for CVD prevention [31].

In this study, compared to the control group, both the average blood pressure values and the incidence of SBP ≥140 mm Hg were significantly higher among internet users with diabetes, in men and women (Table 2). The SBP was mean ± standard deviation 141.7±15.5 mm Hg, and the percentage of internet users with poorly controlled hypertension (SBP ≥140 mm Hg) was 58.7%.

A similar prevalence of arterial hypertension in the population of patients with diabetes was reported in a study from Mulago National Referral Hospital in Uganda. Hypertension in patients with newly diagnosed diabetes was found to be 61.9%, and arterial pressure >140 mm Hg was observed in 51.5% of individuals [32].

The treatment of diabetes, including adherence to dietary recommendations, can be complicated by the presence of anxiety disorders. A 2022 study demonstrated a correlation between the occurrence of anxiety and diabetes. Individuals with anxiety had a 19% increased risk of developing diabetes, while those with diabetes had a 41% increased risk of developing anxiety [33]. This suggests the need for a more comprehensive approach to patients with diabetes, incorporating screening tests for anxiety disorders into the management program.

Individualized comprehensive pharmacological and non-pharmacological treatment of T2DM, taking into account risk factors, could reduce the risk of microvascular and macrovascular complications and improve patient prognosis [34].

## CONCLUSIONS

The diabetic group demonstrated significantly increased SBP and TC levels compared to the non-diabetic control group as well as higher rates of CAD, and smoking in individuals with diabetes mellitus.

These findings underscore the necessity for targeted cardiovascular risk management in the diabetic population.
